# Supplementary material for: Causal relationships between obesity and the leading causes of death in women and men
Source: PLoS Genet. 2019 Oct 24;15(10):e1008405. doi: 10.1371/journal.pgen.1008405 (PMC6812754; doi:10.1371/journal.pgen.1008405)
Supplement: Attachment — Submitted filename: response_to_reviewers_190830.pdf [file pgen.1008405.s003.pdf]

BIG DATA INSTITUTE
Li Ka Shing Centre for Health Information and Discovery

BIG DATA INSTITUTE | UNIVERSITY OF OXFORD

Professor Cecilia Lindgren
Big Data Institute | Li Ka Shing Centre for Health Information and Discovery
Old Road Campus | Oxford | OX3 7LF | United Kingdom
Tel: +44(0)1865287835; Email: cecilia.lindgren@bdi.ox.ac.uk

Oxford, 30th August, 2019

Dear Professor Cotsapas and Professor Tang,

On behalf of all co-authors, we would like to thank you and the two reviewers for the careful review and constructive advice on how to improve our manuscript “Causal relationships between obesity and the leading causes of death in women and men” and for the opportunity to submit a revised version.

We were happy to read the overall positive comments and have now responded to each of the reviewer comments inline and in red. In addition, we have revised the manuscript accordingly, alongside a *track changes* version to facilitate the review process.

We look forward to your future correspondence.

Yours sincerely,

Prof Cecilia M Lindgren, PhD; on behalf of all co-authors

Reviewer's Responses to Questions

Reviewer #1: Overview: In this work, the authors employ genetic risk scores for obesity related traits (body mass index, waist-hip ratio, and BMI-adjusted waist-hip ratio) as instrumental variables in Mendelian Randomization analysis of leading causes of mortality in the UK biobank. The authors accounted for sex in construction of the genetic risk scores and considered potential mediators of mortality risk, e.g smoking and blood pressure traits.
The authors report that obesity causes an increase in risk for many leading causes of death, including coronary artery disease, stroke, chronic obstructive pulmonary disease, lung cancer, type 2 and 1 diabetes mellitus, non-alcoholic fatty liver disease, chronic liver disease and renal failure. For the risk of type 2 diabetes, chronic obstructive pulmonary disease and renal failure, the authors find that the effects of obesity on risk differ between men and women.

Authors' response: We thank the Reviewer for the thorough review.

Comments:
* Except for the number of variants, I could not find information about the exact genetic markers used in the construction of genetic risk scores. It would be useful to have a supplementary data sheet with the annotations of the variants, utilized in specific GRS'.

Authors' response: This is an excellent suggestion. We have now uploaded an excel file that lists all SNPs used in each of the instruments in the main analyses, and refer to them in the manuscript as S1 Table. In addition, all GRSs (including all those used in sensitivity analyses) with the SNPs and weights will be added to GitHub where all of the code will be made available upon publication. We have not included the GRSs used in sensitivity analyses in S1 Table due to the high number of tables this would require (69 tables in total), but hope that you agree that the public availability of GitHub should be sufficient for interested readers. In addition, we have updated the GitHub repository name to reflect the article title (Information added on Page 8, Line 194, Page 14, Line 342-344 and Page 32, Line 932, and "S1 Table" added).

* The sex-specific approach to GRS construction is a potentially useful tool which is very poorly explored in the literature. It could be useful if authors included some information on the utility of the chosen sex-specific GRS-construction approach in and it's possible refinement and future applications in the Discussion section (e. g. under Strength and Weaknesses).

Authors' response: We thank the reviewer for this suggestion. We have added a section about the utility of the sex-specific GRS-construction approach and potential future developments in the Discussion section (Page 24, Lines 597-608).

Reviewer #2: This manuscript addresses the important genetic question of whether the sex dimorphism in obesity-related traits causes a sex difference in risks for complex disorders. To answer this question, the authors apply the Mendelian randomization (MR) technique with sex-specific genetic instrument variables (IV) constructed using published GWAS summary statistics. While I find the sex dimorphism and its potential implication to

pathophysiology can have a major implication to our understanding of the disease genetics, my enthusiasm is diminished by a major concern below.

I am concerned that the proposed method cannot answer how much of the observed sex difference in obesity-mediated disease risk is due to sex dimorphism vs social/environmental differences between sex. For instance, the authors report the sex difference for the risk of being a smoker depending on BMI and WHR. However, how much of this is due to the physiological difference and how much is due to gender norm and social pressure? If it is mostly the latter, the authors can still claim it is genetic - sex is genetically determined, - but my excitement on this work will become diminished. The authors attempt to address this issue in "potential mechanisms" section, but this issue seems to remain largely inconclusive. My concern is not narrowly focused on the issue of smoking status potentially explaining the sex difference in lung disease risks, which is discussed in the Discussion section. Rather, I am concerned that social confounder may affect both exposure (obesity traits) and disease risk and that sex is correlated with this confounder. And this possibility has to be reasonably ruled out in the analyses.

Authors' response: We thank the reviewer for the thoughtful review and the comments.

We agree that the question of whether the effects of adiposity are *mediated* differentially in women vs men by biological (e.g. blood pressure) vs. social (e.g. education) factors is an interesting one. However, provided that such relationships of the obesity instruments with biological or social factors do not represent confounding pathways, this ought to have no impact on the credibility of our findings. Indeed, previous studies have shown that obesity is linked to social factors other than smoking, such as education and income, and that, in keeping with our findings, these may also be differential by sex (1,2). Since these socioeconomic variables are likely to lie on the causal pathway between obesity traits and disease, they ought not confound our findings. While we agree it would be interesting to investigate the extent of mediation from biological vs. social factors, we consider that this falls beyond the scope of the current, already exhaustive, paper.

I am concerned that the CAD did not show strong sex differences. CAD is probably one of the most sex-dimorphic disorders, showing a striking sex difference in the prevalence by age. Is this perhaps because the covariate modeling of age, age^2 and sex not enough to model this prevalence pattern?

Authors' response: We fully agree that CAD prevalence patterns by age differs substantially between men and women, but do not think that this necessarily need to be due to a sex difference in the effect of obesity traits on CAD risk.

There is conflicting evidence from observational epidemiology on whether the association of obesity traits with risk of CAD differs between women and men (3–5). In the studies that do report observational evidence of heterogeneity between women and men, it remains unclear if these differences arise from true sex-differential effects of obesity on risk of CAD or whether they represent bias and/or confounding that is differential by sex. Our findings, using a genetic epidemiological approach that should minimise such sources of bias and confounding, do not show a major difference between adiposity traits and risk of CAD between women and men. Importantly, our findings are consistent with other studies

employing similar designs. For example, the recent study by Lotta *et al.* (6) tested GRSs for various waist- and hip traits (including waist-hip ratio adjusted for BMI) to investigate whether the effect on CAD differed by sex, but did not find any evidence of such a difference – in keeping with our findings. Notably, even though the study by Lotta *et al.* used similar data sources as we did, they used different phenotypes to assess fat distribution and another statistical technique to evaluate sex differences (a P-interaction approach), thus providing a means of validating our findings. Therefore our findings, using statistical approaches that should be less prone to bias and confounding, are in keeping with the contemporary literature and argue against a sex-differential effect of adiposity on risk of CAD.

In regards to the covariate modeling, we only adjusted for sex in the combined-sexes analyses. We note that the current adjustment of age, age^2, and sex has the added benefits of 1) being also used in the original GWAS where we derived our instruments from (7) and 2) make our results more comparable with other studies, as this is one of the most common ways to adjust for age in MR studies involving CAD (e.g. (8)).

I would like to see if Winner's curse in obesity traits did not affect the sex-specific differences. They show that using unweighted GRS does not affect the conclusion, but the unweighted GRS could still be biased toward one sex because of p-value thresholding effect.

Authors' response: We understand this comment to mean that the reviewer is questioning whether winner's curse differs by sex and whether this impacts our findings. To investigate this, we recomputed the MR estimates for the obesity trait-disease combinations where we show evidence of sexual heterogeneity using three additional approaches.

First, we used the sex-combined GWAS to select SNPs for the genetic instrument; such an approach should limit the phenomenon of winner's curse differentially affecting women and men. To do so, we selected SNPs identified in the combined-sexes sample in the original GWAS (7), in which there is an approximately equal number of men and women. After genotype quality control, we weighted the SNPs by the combined-sexes estimates, the sex-specific estimates, or using an unweighted approach giving all SNPs a weight of 1. We then recomputed the MR estimates in men and women for the obesity trait-disease combinations with evidence of sexual heterogeneity. These analyses yielded highly similar results as to the main analyses, and thus provide further evidence of the results being unlikely to arise from winner's curse affecting women and men differently. We have added information and figures relating to the new analyses (Page 14, Lines 338-340 in the main article and Page 9 in the Supplementary Information, and Supplementary Figures G-I, with references to these new analyses and results added on Page 18 Lines 448-449 in the main article). We would also like to note that the use of GIANT 2015-weighted SNPs (9,10) also supported the main findings (Supporting Information, figure D).

Furthermore, the Wald methodology we use to compute our MR estimates mean that the same weights are used in regressions for the exposure and the outcome, before en estimate is computed as the ratio of these (Wald method: $Beta(Y)_{GRS}/Beta(X)_{GRS}$). In effect, this is a division of regressions with a common independent variable, meaning that differential weights should not cause notable differences in the MR estimates. In addition, we also used

a strict Bonferroni-corrected P-value threshold as evidence in support of sexual heterogeneity to limit spurious findings.

Finally, bias from winner's curse decreases with increased power, as estimates become more precise (11–13). The large sample sizes (315,284-806,834) in the GWAS (7) we derived our instruments in should therefore also limit bias from winner's curse.

However, we have also added this as a potential limitation in the Discussion section of the main article (Page 23, Lines 582-58).

General changes

In addition to the changes detailed above, we have:

1. Added that Professor Lindgren has collaborated with Bayer in research in the online disclosure from. This is a new disclosure that was not present at the initial submission.
2. We have changed the Bycroft *et al.* reference to the published article, as we previously incorrectly referenced a pre-print version in both the main article (reference 23, Page 26, Lines 692-693) and the supplement (reference 16, Page 60). In addition, we have added the Sudlow *et al.* reference (reference number 22, Page 26, Lines 689-691) for the UK Biobank for accuracy (Page 7, Lines 152 and 169; Page 8, Line 194; Page 9, Line 202) and updated the other reference numbers accordingly.
3. We have added that the smoking status phenotype derivation was performed using baseline information only for correctness (Page 11, Line 257).
4. We have added a reference in regards to the effect of obesity traits on CAD that had been accidentally deleted during the formatting process (Dhana *et al.*, reference number 78) (Reference added on Page 20, Line 514, actual reference on Page 29 Lines 830-832).
5. We have removed the word "Finally, " to improve flow (Page 23, Line 591).
6. We have removed the funding and the disclosures from the Acknowledgements section in response to a suggestion from the editorial team (Page 24, Lines 617 and 619).
7. We have also updated the table of contents on Page 1-2 in the Supplementary Information to reflect the added information and figures.
8. In response to a suggestion from the editorial team, we would like to highlight that figure 4 only shows results from the Mendelian randomization analyses. The diseases not shown on the plot were only investigated using regression analyses and not Mendelian randomization, and hence cannot be included in the plot. We have, however, updated the figure legend for figure 4 in the main manuscript with a reference to the Supplementary table where the regression results can be found in response to this (Page 16-17, Lines 408-409). In summary, all the referenced results are available in the manuscript and supplementary tables.

References

1. Howe LD, Kanayalal R, Beaumont RN, Davies AR, Frayling TM, Harrison S, et al. Effects of body mass index on relationship status, social contact, and socioeconomic position: Mendelian Randomization study in UK Biobank [Internet]. bioRxiv. 2019 [cited 2019 Aug 21]. p. 524488. Available from: https://www.biorxiv.org/content/10.1101/524488v1

2. Tyrrell J, Jones SE, Beaumont R, Astley CM, Lovell R, Yaghootkar H, et al. Height, body mass index, and socioeconomic status: mendelian randomisation study in UK Biobank. BMJ. 2016 Mar 8;352:i582.

3. Dagenais GR, Yi Q, Mann JFE, Bosch J, Pogue J, Yusuf S. Prognostic impact of body weight and abdominal obesity in women and men with cardiovascular disease. Am Heart J. 2005 Jan 1;149(1):54–60.

4. Rost S, Freuer D, Peters A, Thorand B, Holle R, Linseisen J, et al. New indexes of body fat distribution and sex-specific risk of total and cause-specific mortality: a prospective cohort study. BMC Public Health. 2018 Dec 2;18(1):427.

5. Dhana K, Kavousi M, Ikram MA, Tiemeier HW, Hofman A, Franco OH. Body shape index in comparison with other anthropometric measures in prediction of total and cause-specific mortality. J Epidemiol Community Health. 2016 Jan;70(1):90–6.

6. Lotta LA, Wittemans LBL, Zuber V, Stewart ID, Sharp SJ, Luan J, et al. Association of Genetic Variants Related to Gluteofemoral vs Abdominal Fat Distribution With Type 2 Diabetes, Coronary Disease, and Cardiovascular Risk Factors. JAMA. 2018 Dec 25;320(24):2553.

7. Pulit SL, Stoneman C, Morris AP, Wood AR, Glastonbury CA, Tyrrell J, et al. Meta-analysis of genome-wide association studies for body fat distribution in 694 649 individuals of European ancestry. Hum Mol Genet. 2019 Jan 1;28(1):166–74.

8. Sun Y-Q, Burgess S, Staley JR, Wood AM, Bell S, Kaptoge SK, et al. Body mass index and all cause mortality in HUNT and UK Biobank studies: linear and non-linear mendelian randomisation analyses. BMJ. 2019 Mar 26;364:l1042.

9. Locke AE, Kahali B, Berndt SI, Justice AE, Pers TH, Day FR, et al. Genetic studies of body mass index yield new insights for obesity biology. Nature. 2015 Feb 12;518(7538):197–206.

10. Shungin D, Winkler TW, Croteau-Chonka DC, Ferreira T, Locke AE, Mägi R, et al. New genetic loci link adipose and insulin biology to body fat distribution. Nature. 2015 Feb 12;518(7538):187–96.

11. Huang QQ, Ritchie SC, Brozynska M, Inouye M. Power, false discovery rate and Winner's Curse in eQTL studies. Nucleic Acids Res. 2018 Dec 14;46(22):e133.

12. Zollner S, Pritchard JK. Overcoming the winner's curse: estimating penetrance parameters from case-control data. Am J Hum Genet. 2007 Apr;80(4):605–15.

13. Marigorta UM, Rodríguez JA, Gibson G, Navarro A. Replicability and Prediction: Lessons and Challenges from GWAS. Trends Genet. 2018 Jul;34(7):504–17.
